# Early Detection of Vulnerable Plaques Using Targeted Biosynthetic Nanobubbles

**DOI:** 10.3390/ph18091285

**Published:** 2025-08-28

**Authors:** Yan Wang, Huang Yin, Rui Zhang, Dan Yu, Jieqiong Wang, Tingting Liu, Xiong Shen, Li Xue, Fei Yan

**Affiliations:** 1 Department of Cardiovascular Ultrasound, The Fourth Affiliated Hospital of Harbin Medical University, Harbin 150001, China; hydwyan@163.com (Y.W.); zhangr_1995@126.com (R.Z.); 15005054415@163.com (D.Y.); 2 The Fifth Affiliated Hospital, Sun Yat-sen University, Zhuhai 519000, China; huangyin3@sysu.edu.cn; 3 Department of Rehabilitation Medicine, Huashan Hospital, Fudan University, Shanghai 201206, China; wangjieqiong.123@foxmail.com; 4 Department of Ultrasound, The Second People’s Hospital of Shenzhen, The First Affiliated Hospital of Shenzhen University, Shenzhen 518061, China; ltingting949@gmail.com (T.L.); shenxiong2023@126.com (X.S.); 5 Ultrasonic Medicine, Graduate School, Guangxi University of Chinese Medicine, Nanning 530200, China; 6 Heilongjiang Provincial Molecular Medicine Engineering Technology Research Center, Harbin 150001, China; 7 State Key Laboratory of Quantitative Engineering Biology, Shenzhen Institute of Synthetic Biology, Shenzhen Institutes of Advanced Technology, Chinese Academy of Sciences, Shenzhen 518055, China

**Keywords:** vulnerable plaque, ultrasound molecular imaging, gas vesicles, biosynthetic nanobubbles

## Abstract

**Objectives:** Atherosclerosis is a chronic inflammatory disease characterized by complex pathological mechanisms. Early detection of vulnerable plaques is critical for assessing rupture risk and preventing acute cardiovascular events. Conventional ultrasound contrast agents (UCAs) are limited in their ability to penetrate the vascular wall and unable to provide detailed information on plaque composition and stability. In this study, we developed biosynthetic gas vesicles (GVs) derived from *Halobacterium* NRC-1 as UCAs for imaging of vulnerable plaques. **Methods:** These GVs were functionalized with the VHPKQHR peptide (VHP), enabling specific binding to vascular cell adhesion molecule-1 (VCAM-1), a key biomarker of inflammation in atherosclerosis. In vitro evaluation of VHP-GVs was performed through contrast-enhanced ultrasound imaging using agarose gel phantoms and adhesion assays with inflammatory cell models to assess their targeting capability toward VCAM-1. In vivo ultrasound molecular imaging was performed using the Sprague Dawley (SD) rat model of early-stage atherosclerosis in the left common carotid artery to evaluate imaging efficacy. **Results:** Both in vitro and in vivo experiments demonstrated that VHP-GVs could effectively penetrate the vascular wall into plaques and generate robust ultrasound contrast signals for precise identification of vulnerable regions. **Conclusions:** This study establishes a promising tool for the early diagnosis and targeted treatment of atherosclerosis, underscoring the translational potential of biosynthetic nanobubbles in clinical practice.

## 1. Introduction

Atherosclerosis is initiated by the accumulation of oxidized low-density lipoprotein (oxLDL), which triggers endothelial cell activation and upregulation of adhesion molecules, such as intercellular adhesion molecule-1 (ICAM-1), VCAM-1, and P-selectin, etc. [1,2]. Among these, VCAM-1 plays a pivotal role in plaque formation by mediating monocyte adhesion and migration into the vessel wall [3]. As an early inflammatory marker, VCAM-1 represents a valuable target for the non-invasive detection of vulnerable plaques [4,5]. Studies have demonstrated that plaque stability is closely associated with the degree of inflammation, elevated expression of inflammatory factors correlating with increased plaque instability, and rupture risk [6]. When the calcium in the plaque gradually increased, the plaque gradually stabilized [7]. Therefore, early identification and precise characterization of these plaques are critical for effective intervention and management [8].

With the development of molecular imaging technology, non-invasive imaging techniques commonly used for diagnosing atherosclerotic plaques often attempt to enhance detection sensitivity and specificity through targeted nanoscale molecular imaging probes [2,9]. Ultrasound molecular imaging technology (UMI) allows for dynamic monitoring of markers, possesses real-time imaging capabilities, and exhibits high tissue penetration [10]. The most common ultrasound probe is a micrometer-sized bubble with a gas core encapsulated in a biocompatible shell, eliminating the limitations of radiation exposure and nephrotoxicity [11]. Compared to computed tomography (CT), magnetic resonance imaging (MRI), and positron emission tomography (PET), ultrasound has become an important tool for plaque screening due to its advantages, such as non-radiation, real-time dynamic observation, high cost-effectiveness, and repeatability of examination [12,13].

Microbubble-based acoustic probes have been designed to image key biomarkers associated with vulnerable plaques [14]. Existing studies on UCAs include the assessment of plaque stability by interfering with the inflammatory response within the plaque by microbubble delivery of IL-8 antibodies [15], targeted imaging of plaques using hyaluronic acid-conjugated ligands targeting the highly exclusionary cellular receptor CD44 on the surface of the plaque [16], and the preparation of triple-targeted microbubbles UCA labeled with multiple inflammatory targets to significantly enhance ultrasound imaging signals by reducing off-target effects [17], etc. However, the size of conventional microbubbles (1–10 µm) limits their ability to penetrate the endothelial barrier [10,18], thus hindering the acquisition of detailed information on plaque composition. Recent advancements in nanotechnology have facilitated the development of nanoscale UCAs [19], such as acoustic liposomes [20], perfluorocarbon nanodroplets [20,21], silica nanoparticles [22], and polymeric particles [23]. Nevertheless, most of these agents are chemically synthesized, facing challenges related to stability, particle size distribution, and toxic side effects [24]. Some polymers have poor ultrasound visualization (e.g., polymeric vesicles and polymeric hydrogels) or need to be activated in vivo through special routes to produce ultrasound signals, which increases the complexity of operation and equipment requirements [23]. Recent studies have demonstrated that spindle-shaped gas vesicles (GVs) can be isolated from *Halobacterium salinarum* NRC-1 [25,26,27]. These biosynthetic nanoscale GVs consist of hollow protein shells filled with air-like gases and have shown excellent ultrasound contrast signals in murine liver and tumor models [26,28].

**Figure 1:**
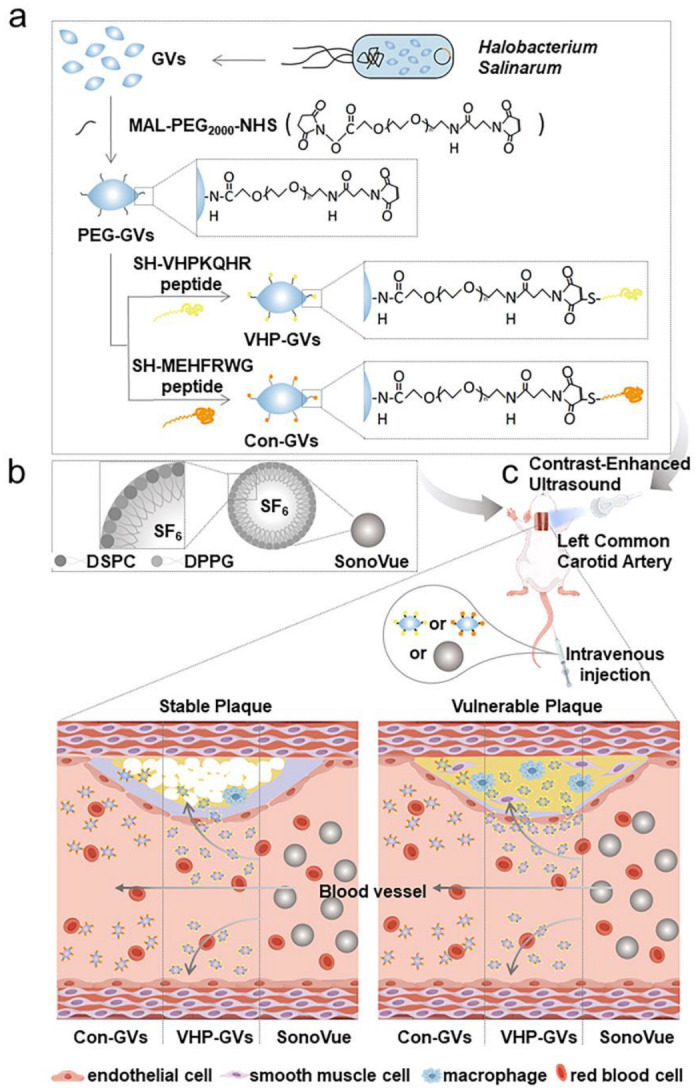
Schematic diagram of biosynthesis and ultrasound molecular imaging (UMI) of GVs for in vivo assessment of atherosclerotic plaques. (**a**) Modification of GVs isolated from *Halobacterium salinarum* NRC-1 with MAL-PEG_2000_-NHS, VHPKQHR peptide (VHP), and MEHFRWG peptide (Con) to produce VHP-GVs and Con-GVs. (**b**) Schematic representation of SonoVue structure. (**c**) Assessment of UMI using VHP-GVs for detecting early-stage atherosclerotic vulnerable plaques. VHP-GVs, Con-GVs, and SonoVue were individually administered to Sprague Dawley (SD) rats with early atherosclerotic plaques. Owing to their nanoscale dimensions, VHP-GVs efficiently traversed the vascular endothelium and bound to VCAM-1 expressed on plaque cells, generating UMI signals at the plaque site. In contrast, SonoVue remained confined to the vascular lumen, producing ultrasonographic signals exclusively within this region.

In this study, we developed a kind of highly specific targeting probe (VHP-GVs) through conjugating the linear peptide VHPKQHR onto the surface of GVs for the molecularly imaging of atherosclerotic plaques using UMI. The VHP peptide shares structural homology with the ligand of VCAM-1 and has been confirmed to exhibit high affinity for VCAM-1 [29,30,31,32,33,34]. As the control, the linear peptide with the MEHFRWG sequence was synthesized and conjugated onto the surface of GVs by using the same strategy (Con-GVs). Through comparing the control peptide with the VHP peptide in contrast imaging studies, we were able to exclude the nonspecific effects of short-chain peptides on imaging contrast (Figure 1). In this study, we successfully functionalized GVs with the VHP peptide to create VCAM-1-targeted nanobubbles. Through comprehensive in vitro and in vivo ultrasonographic imaging analyses, we evaluated the diagnostic potential of these biosynthetic nanobubbles for vulnerable plaques.

## 2. Results

### 2.1. Characterization Results of Gas Vesicles

**Figure 2:**
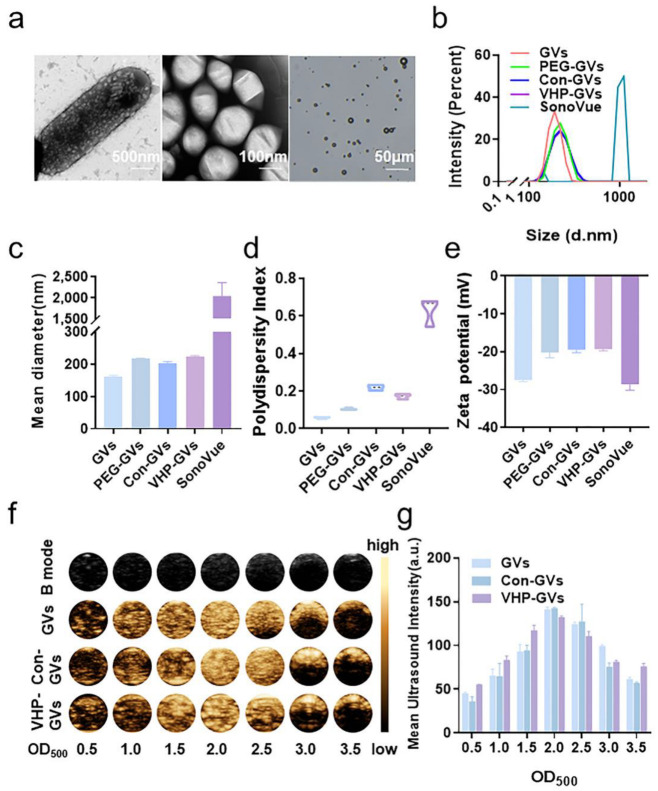
Characterization and ultrasound imaging of gas vesicles (GVs) in vitro. (**a**) TEM images of *Halobacterium salinarum* NRC-1 (**left**) and isolated GVs (**middle**), along with SonoVue imaged under the bright field of an inverted fluorescence microscope (**right**). Scale bars represent 500 nm, 100 nm, and 50 µm from left to right, respectively. (**b**) Size distributions of GVs, Con-GVs, VHP-GVs, and SonoVue. (**c**) Average diameters of GVs, Con-GVs, VHP-GVs, and SonoVue. (**d**) PDI of GVs, Con-GVs, VHP-GVs, and SonoVue. (**e**) Zeta potentials of GVs, Con-GVs, VHP-GVs, and SonoVue. (**f**) In vitro ultrasonography imaging of GVs, Con-GVs, and VHP-GVs at varying concentrations (OD_500_ = 0.5–3.5). (**g**) Quantitative analysis of the average ultrasonic signal intensity from (**f**). Data in (**c**–**e**,**g**) are presented as mean ± standard deviation from three independent experiments.

GVs were extracted and purified from *Halobacterium* using a previously established protocol (Figure S1). Transmission electron microscopy (TEM) imaging of *Halobacterium* revealed the presence of abundant intracellular vesicle-structure GVs (Figure 2a, left). Isolated GVs displayed a uniform fusiform morphology, with a long diameter of approximately 200–300 nm and a short diameter of 100–200 nm (Figure 2a, middle). In contrast, SonoVue particles exhibited a spherical morphology with variable sizes, ranging from 1 to 10 μm in diameter (Figure 2a, right). Particle size analysis using a Zetasizer demonstrated a narrow size distribution for GVs (Figure 2b), with an average diameter of 161.47 ± 3.56 nm and a polydispersity index (PDI) of 0.06 ± 0.004 (Figure 2c,d). After maleimide polyethylene glycol-activated ester (MAL-PEG_2000_-NHS, abbreviated as PEG) modification and peptide conjugation, the size of GVs increased slightly but remained within a relatively narrow distribution, with PEG-conjugated GVs (PEG-GVs) at 217.43 ± 1.45 nm (PDI = 0.11 ± 0.005), Con-GVs at 203.90 ± 4.25 nm (PDI = 0.22 ± 0.01), and VHP-GVs at 222.10 ± 3.56 nm (PDI = 0.06 ± 0.01). In comparison, SonoVue exhibited a significantly larger particle size of 2045.67 ± 315.97 nm and a broader size distribution (PDI = 0.63 ± 0.08; Figure 2b–d). Zeta potential analysis revealed that both GVs and SonoVue carried a net negative charge, with values of −27.47 ± 0.45 mV and −28.6 ± 1.61 mV, respectively. PEGylation of GVs increased the zeta potential of GVs to −20.20 ± 1.39 mV, while subsequent peptide conjugation resulted in minimal changes (Con-GVs = −19.47 ± 0.84 mV; VHP-GVs = −19.37 ± 0.42 mV), indicating similar surface charge properties across modified GVs (Figure 2e). Stability assessment results demonstrated no significant differences (*p* > 0.05) in particle size, PDI, or zeta potential for various GVs between freshly prepared samples and those stored for 3 or 7 days in PBS buffer, indicating the stability of GVs could be maintained for at least 7 days (Figure S2a–c). In addition, further experimental results indicate that VHP-GVs maintain good stability without significant changes in characterization when stored in PBS, cell culture medium, and serum for 7 days (Figure S2d–f).

### 2.2. In Vitro Contrast-Enhanced Ultrasound Imaging Results of GVs

To evaluate the contrast imaging performance of the three types of GVs, the in vitro ultrasonography was performed using agar phantom wells with various concentrations of GVs, Con-GVs, and VHP-GVs (OD_500_ from 0.5 to 3.5). For GV concentrations between OD_500_ 0.5 and OD_500_ 2.5, the contrast signals of GVs, Con-GVs, and VHP-GVs increased progressively with the increase in concentrations. However, when higher concentrations (OD_500_ 2.5 and above) were used, the images appeared as anterior crescent shape, with significant posterior attenuation. At equivalent concentrations, GVs, VHP-GVs, and Con-GVs exhibited similar contrast signal intensities (Figure 2f,g).

### 2.3. In Vitro Cell Targeting of VHPs and VHP-GVs

**Figure 3:**
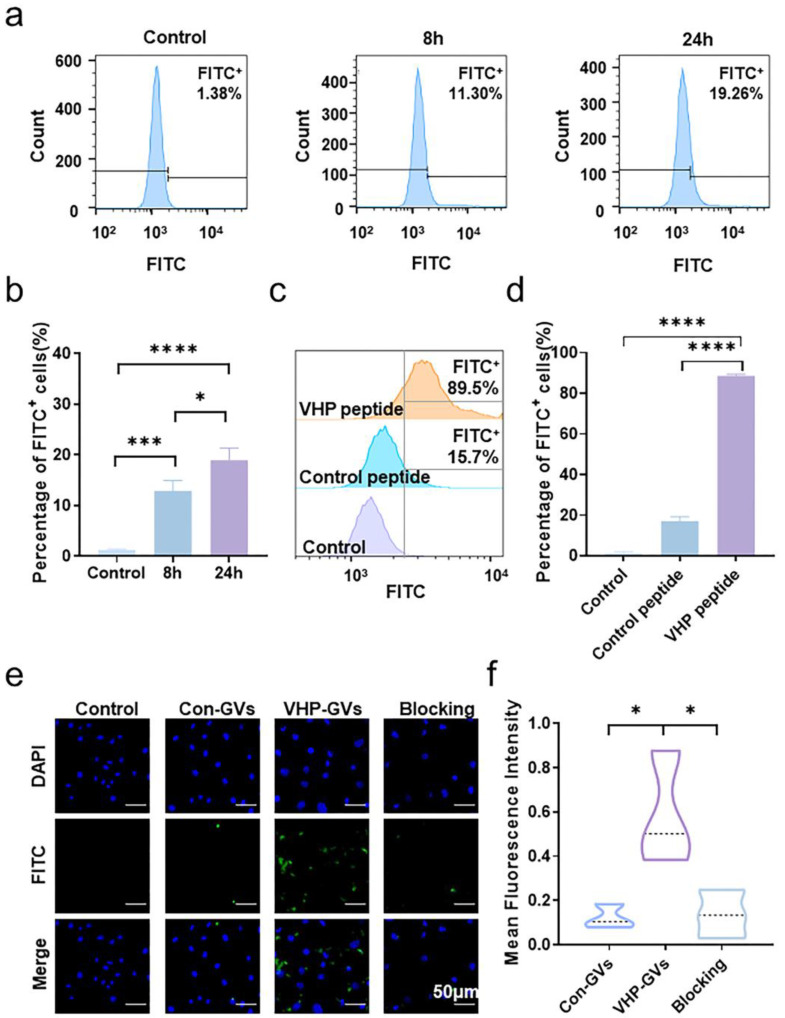
Cell model validation and in vitro targeting ability of the VHP peptide and VHP-GVs. (**a**) Flow cytometric analysis of expression levels of VCAM-1 on the surface of HUVECs after 8 h and 24 h by TNF-α stimulation. FITC^+^ stands for FITC-positive. The percentages of FITC-positive cells after 8 h and 24 h of TNF-α stimulation were 11.3% and 19.26%, respectively. (**b**) Quantitative analysis of the percentages of FITC-positive cells in (**a**). (**c**) Flow cytometric analysis of the binding capacity of VHP peptides to TNF-α-stimulated HUVECs in vitro. FITC^+^ stands for FITC-positive. (**d**) Quantitative analysis of the percentages of FITC-positive cells in (**c**). (**e**) In vitro binding of VHP-GVs to TNF-α-stimulated HUVECs. Representative fluorescence microscopy images show HUVECs incubated with FITC-labeled VHP-GVs, FITC-labeled Con-GVs, and a control group pre-blocked with free VHP peptides followed by incubation with FITC-labeled VHP-GVs. FITC-labeled GVs are shown in green, and DAPI-stained nuclei are shown in blue. Scale bar = 50 µm. (**f**) Quantitative analysis of fluorescence intensity from (**e**). Data in (**b**,**d**,**f**) are presented as mean ± standard deviation from three independent experiments. Statistical significance levels are denoted as * for *p* < 0.05, *** for *p* < 0.001 and **** for *p* < 0.0001.

Flow cytometry analysis revealed that stimulation of human umbilical vein endothelial cells (HUV-EC-Cs, HUVECs) with tumor necrosis factor-α (TNF-α) at a concentration of 40 ng/mL for either 8 (11.30%, *p* < 0.001) or 24 h (19.26%, *p* < 0.0001) significantly increased the expression level of VCAM-1 compared to unstimulated cells (1.38%). Notably, VCAM-1 expression after 24 h of TNF-α-stimulation was further elevated in comparison to 8 h of TNF-α-stimulation (*p* < 0.05), indicating that 24 h of TNF-α stimulation was more effective in establishing a robust cellular inflammation model (Figure S3 and Figure 3a,b). To assess the cell affinity of the VHP peptide, HUVECs were stimulated with TNF-α (40 ng/mL) for 24 h and subsequently co-incubated with the fluorescein isothiocyanate (FITC)-labeled VHP peptide or the FITC-labeled control peptide. Flow cytometry analysis demonstrated that the fluorescence intensity of HUVECs incubated with an FITC-labeled VHP peptide was significantly higher (89.5%, *p* < 0.0001) than that of the blank control group, while the control peptide-incubated HUVECs exhibited only a modest increase (15.7%) in fluorescence (Figure S4 and Figure 3c,d). These results suggest that the VHP peptide but not the control peptide can bind to HUVECs. We provided the flow cytometry gating strategy for expression levels of VCAM-1 on the surface of HUVECs after 8 h and 24 h by TNF-α stimulation (Figure S3) and VCAM-1 targeting experiments (Figure S4). Debris was excluded based on SSC-A vs. FSC-A. Single cells were selected from FSC-H vs. FSC-A. FITC-positive cells were defined relative to a cell-only control sample. Next, the cell adhesion with Con-GVs and VHP-GVs probes was evaluated. Laser confocal microscopy revealed abundant green fluorescence surrounding the nuclei of HUVECs in the VHP-GVs group, indicating robust adhesion of VHP-GVs to the cell surface. In contrast, minimal fluorescence signals were observed in the blocking (the experimental group that is pre-treated with the VHP peptide to preemptively occupy the binding sites for VHP-GVs before detecting the binding ability of VHP-GVs at this time) and Con-GVs groups (Figure 3e). Quantitative analysis of fluorescence intensity confirmed that the VHP-GVs group exhibited significantly higher fluorescence intensity than the Con-GVs and blocking groups (*p* < 0.05; Figure 3f).

### 2.4. In Vivo Ultrasound Molecular Imaging Results

**Figure 4:**
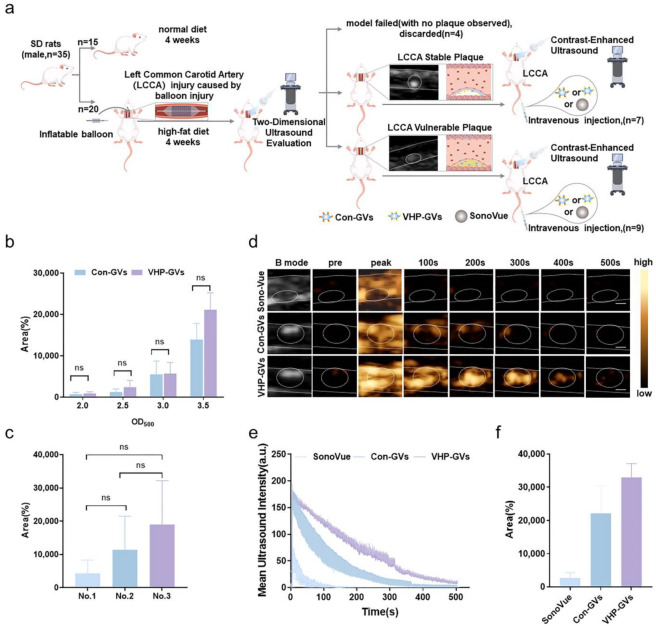
In vivo ultrasound molecular imaging in SD rats. (**a**) Schematic diagram of preparation, grouping, and experimental operation of atherosclerotic plaque rat models. The two white dashed lines are used to indicate the vascular wall lumen, and white circles are used to indicate the observed plaque site in the ultrasound legend. (**b**) In vivo contrast imaging of Con-GVs and VHP-GVs at varying concentrations (OD_500_ = 2.0, 2.5, 3.0, 3.5) in SD rats. Area differences under the contrast signal intensity curves (ΔAUC) of Con-GVs and VHP-GVs were measured 500 s after intravenous injection. (**c**) The ΔAUC of the same SD rat injected three times with the same concentration of PEG-GVs was measured over 500 s. (**d**–**f**) In vivo ultrasound molecular imaging of SD rats with atherosclerotic plaques. (**d**) Nonlinear contrast images of SonoVue, Con-GVs, and VHP-GVs (OD_500_ = 3.5) at different time points after intravenous injection. (The contrast signal peaks were observed at the 5th second for SonoVue and at the 3rd second for Con-GVs and VHP-GVs after intravenous injection.) The two white dashed lines are used to indicate the vascular wall lumen, and white circles are used to indicate the observed plaque site. Scale bar = 3000 µm. (**e**) Temporal intensity curves of SonoVue, Con-GVs, and VHP-GVs in the region of interest (ROI) of atherosclerotic plaques after intravenous injection. (**f**) Comparison of the signal intensities of SonoVue, Con-GVs, and VHP-GVs at 500 s post-injection using ΔAUC. Data are presented as mean ± standard deviation from three independent experiments, and ns for no statistical significance.

The in vivo ultrasound molecular imaging capabilities of Con-GVs and VHP-GVs were evaluated in random Sprague Dawley (SD) rats with atherosclerotic plaques (Figure 4a). When Con-GVs and VHP-GVs were injected, no statistically significant differences in overall imaging signals (including the lumen and wall of blood vessel) were observed between the two groups at the same concentration (Figure 4b, Figures S5 and S6). The contrast imaging signals of Con-GVs and VHP-GVs improved progressively with the increase in injection dose, with optimal imaging signals at OD_500_ = 3.5. Therefore, this concentration at OD_500_ = 3.5 was selected for subsequent experiments in SD rats with plaques.

To accurately assess the in vivo ultrasound molecular imaging differences between Con-GVs and VHP-GVs at the same lesion site, a sequential injection protocol was employed. VHP-GVs were first injected, followed by Con-GVs after the initial imaging signals had completely cleared. This approach minimizes the impact of the second-dose enhancement phenomenon, which arises from the saturation of mononuclear phagocyte system uptake following the initial dose (Figure 4c, Figure S7 and S8).

Next, SD rats with atherosclerotic plaques in the left common carotid artery were injected with SonoVue, Con-GVs, and VHP-GVs for ultrasound examination. The experimental results of ultrasound contrast imaging with different contrast agents in rats with atherosclerotic plaques are obtained. Interestingly, SonoVue reached peak contrast signal intensity at 5 s post-injection, filling in the lumen, with contrast signal defects at plaque sites. Subsequently, the intraluminal contrast signal intensity rapidly attenuated and completely cleared within 200 s. By contrast, Con-GVs and VHP-GVs demonstrated significant enhancement of the vascular wall, especially at plaque sites, with peak signal intensity within 3–5 s post-injection. As the time extended, the overall signal intensities gradually decreased (Figure 4d–f).

**Figure 5:**
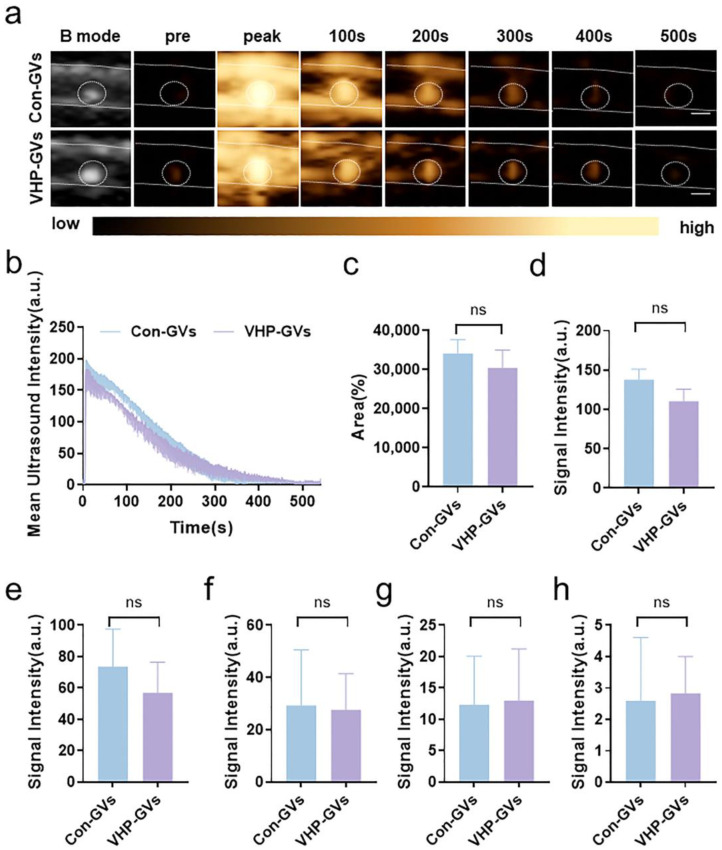
In vivo ultrasound molecular imaging of stable plaques. (**a**) Nonlinear contrast images of Con-GVs and VHP-GVs (OD_500_ = 3.5) at different time points after intravenous injection. (The contrast signal peaks were observed at the 3rd second for Con-GVs and VHP-GVs after intravenous injection.) The two white dashed lines are used to indicate the vascular wall lumen, and white circles are used to indicate the observed plaque site. Scale bar = 3000 µm. (**b**) Temporal intensity curves of Con-GVs and VHP-GVs in the ROI of atherosclerotic stable plaques after intravenous injection. (**c**) Comparison of the signal intensities of Con-GVs and VHP-GVs within 500 s post-injection using ΔAUC. (**d**–**h**) Contrast signals of Con-GVs and VHP-GVs in stable plaques at (**d**) 100 s, (**e**) 200 s, (**f**) 300 s, (**g**) 400 s, and (**h**) 500 s. Data are presented as mean ± standard deviation from three independent experiments, and ns for no statistical significance.

**Figure 6:**
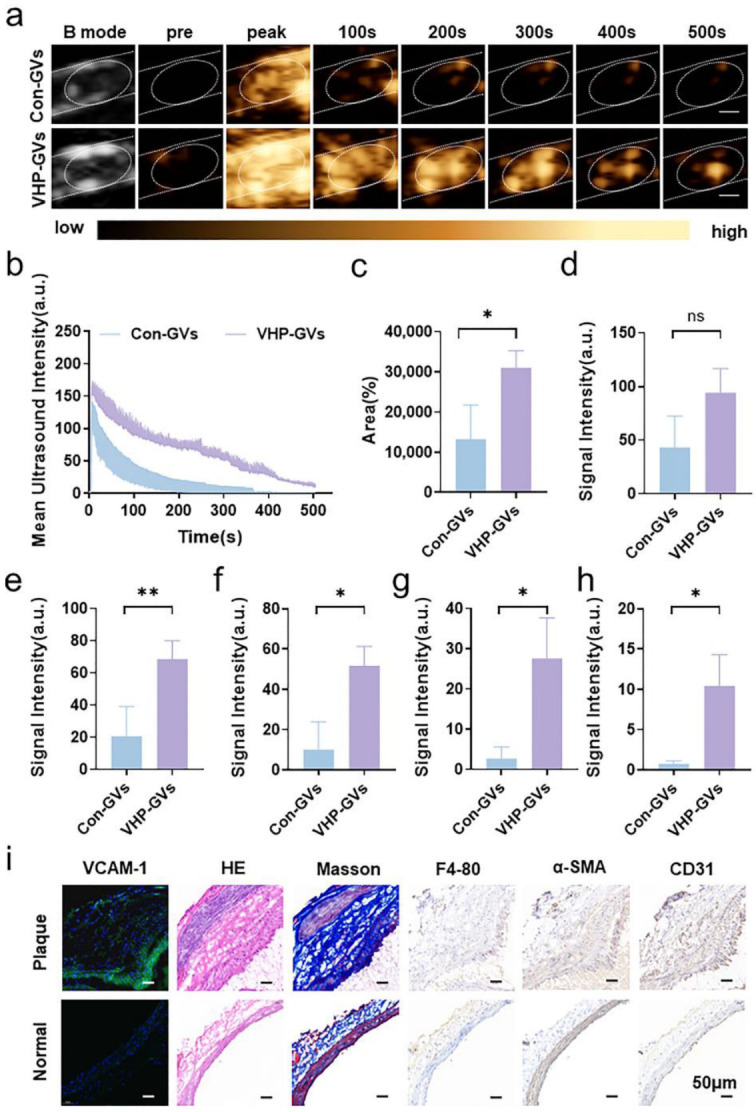
In vivo ultrasound molecular imaging and histologic examination of atherosclerotic vulnerable plaques. (**a–h**) In vivo ultrasound molecular imaging of atherosclerotic vulnerable plaques. (**a**) Nonlinear contrast images of Con-GVs and VHP-GVs (OD_500_ = 3.5) at different time points after intravenous injection. (The contrast signal peaks were observed at the 3rd second for Con-GVs and VHP-GVs after intravenous injection.) The two white dashed lines are used to indicate the vascular wall lumen, and white circles are used to indicate the observed plaque site. Scale bar = 3000 µm. (**b**) Temporal intensity profiles of Con-GVs and VHP-GVs in the ROI of atherosclerotic vulnerable plaques after intravenous injection. (**c**) Comparison of the signal intensities of Con-GVs and VHP-GVs within 500 s post-injection using ΔAUC. (**d**–**h**) Contrast signals of Con-GVs and VHP-GVs in vulnerable plaques at (**d**) 100 s, (**e**) 200 s, (**f**) 300 s, (**g**) 400 s, and (**h**) 500 s. (**i**) Histologic examination of atherosclerotic vulnerable plaques. Immunofluorescence results show VCAM-1 in green and DAPI-stained nuclei in blue. Immunohistochemical results show hematoxylin-stained nuclei in blue and DAB-positive expression in tan. Masson staining reveals collagen fibers in blue and myofibers in red. Scale bar = 50 µm. Data are presented as mean ± standard deviation from four independent experiments. Statistical significance levels are denoted as * for *p* < 0.05, and ** for *p* < 0.01.

Notably, different imaging performance of Con-GVs and VHP-GVs was observed across plaque phenotypes, as evidenced by quantitative contrast analysis (Figure 5 and Figure 6). For stable plaques with significant calcified hyperechoic signals in two-dimensional ultrasound mode, contrast-enhanced ultrasound images at different time points post-injection are shown in Figure 5a. Both Con-GVs and VHP-GVs exhibited contrast signal enhancement at plaque sites. However, the time-intensity curves showed a consistent downward trend (Figure 5b). The differences in contrast signal intensity at stable plaque sites within 500 s (Figure 5c–h) were not statistically significant (*p* > 0.05).

However, for vulnerable plaques exhibiting hypoechoic or mixed echogenicity, or those not detectable in two-dimensional ultrasound mode, VHP-GVs exhibited significantly stronger contrast signal enhancement at plaque sites than that of Con-GVs (Figure 6a). The time-intensity curves showed that VHP-GVs had a slower decline in contrast signal intensity at the plaque site compared to Con-GVs (Figure 6b). Within 500 s after injection, VHP-GVs exhibited significantly higher signal intensity area under curve (AUC) at the vulnerable plaque site compared to Con-GVs (*p* < 0.05; Figure 6c). Quantitative analysis of the contrast signal intensity differences in the vulnerable plaque were significantly higher in the VHP-GVs group than those of Con-GVs at the 200, 300, 400, and 500 s time point, (all *p* < 0.05, and *p* < 0.01 for the 200 s time point; Figure 6d–h). Moreover, VHP-GVs demonstrated selective targeting of plaques, with complete signal attenuation at non-lesion sites, confirming their superior imaging performance for vulnerable plaques. Histological analysis of frozen sections from vulnerable plaque lesions revealed high level expression of VCAM-1 on the endothelium protruding into the lumen, which is consistent with the ultrasonographic findings. During the experiment, it was observed that after injecting VHP-GVs, the imaging time at the vulnerable plaque site could be extended to 16 min before subsiding. The comprehensive image of contrast agent metabolism in Figure S9. The image shows that the blood half-life of VHP-GVs (estimated based on the ultrasound signal intensity of the contrast agent) is reached at 180 s after injection (Figure S9).

Immunohistochemical staining and Masson’s trichrome staining showed proliferative infiltration of macrophages and smooth muscle cells, predominantly smooth muscle cells, accompanied by collagen fibers. The presence of neovascularization was confirmed by CD31 staining, aligning with the characteristics of vulnerable plaques [35] (Figure 6i). All of these data demonstrated that VHP-GVs but not Con-GVs can molecularly image vulnerable plaque lesions.

The repeatability test results show that the ultrasound signal intensity of PEG-GVs, as well as Con-GVs and VHP-GVs in calcified plaques and vulnerable plaques, exhibited good consistency between observers and within observers (Table S1 in the Supplementary Materials).

### 2.5. Biosafety Assessment

**Figure 7:**
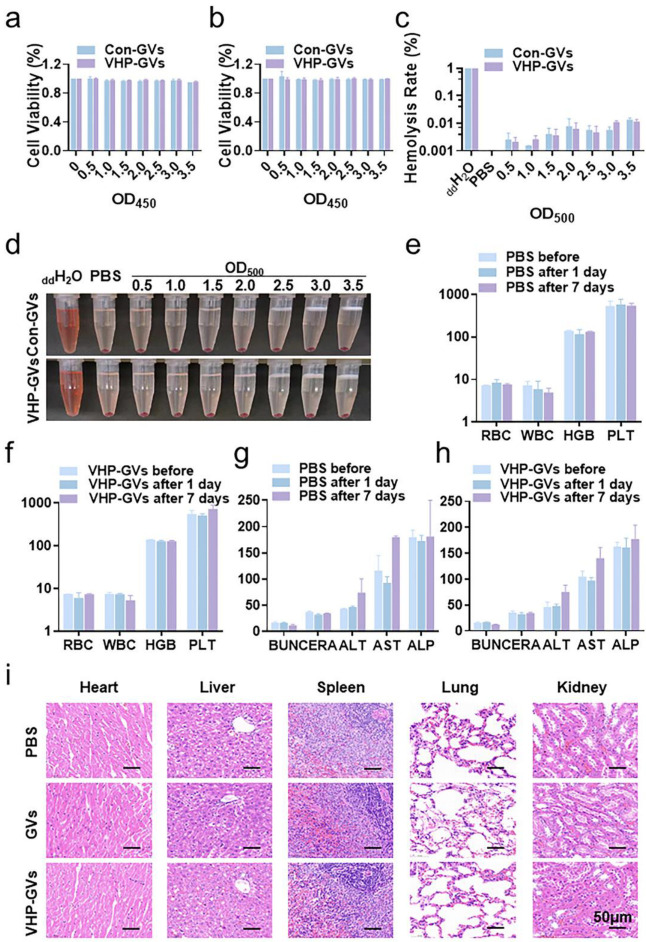
Biosafety analysis. (**a**,**b**) Cell viability assay of HUVECs treated with VHP-GVs or Con-GVs at concentrations of OD_500_ 0–3.5 for 24 h (**a**) and 48 h (**b**). (**c**,**d**) Hemolysis assay of VHP-GVs at OD_500_ 0.5–3.5, with distilled water (ddH_2_O) as a positive control and PBS as a negative control. (**e**,**f**) Blood routine index levels in rats after injection of an equal volume of PBS (**e**) or VHP-GVs (**f**). RBC was expressed as 10^12^/L; WBC and PLT were expressed as 10^9^/L; HGB was expressed as g/L. (**g**,**h**) Liver and renal function indexes in rats after injection of an equal volume of PBS (**g**) or VHP-GVs (**h**). ALT, AST, and ALP were expressed as U/L; BUN and CREA were expressed as mg/dL and μmol/L, respectively. (**i**) Representative H&E-stained sections of major organs (heart, liver, spleen, lungs, kidneys) from rats 7 days after injection of PBS, GVs, or VHP-GVs. Scale bar = 50 μm. Data are presented as mean ± standard deviation from three independent experiments.

In vitro cytotoxicity was evaluated by incubating HUVECs with various concentrations of Con-GVs and VHP-GVs for 24 h or 48 h, followed by the Cell Counting Kit-8 (CCK-8) assay. The results indicated that both Con-GVs and VHP-GVs did not exhibit significant cytotoxicity to HUVECs at all tested concentrations after 24 h (Figure 7a) or 48 h (Figure 7b), similar to the PBS control. The hemolysis analysis showed that neither VHP-GVs nor Con-GVs induced hemolysis at these tested concentrations (OD_500_ = 0.5, 1.0, 1.5, 2.0, 2.5, 3.0, and 3.5) (Figure 7c,d).

To evaluate the in vivo biosafety of VHP-GVs, healthy SD rats were administered intravenous injections of VHP-GVs or PBS. Blood samples collected before injection, on Day 1, and on Day 7 post-injection. Our data revealed no significant changes in routine blood indices, including white blood cells (WBCs), red blood cells (RBCs), hemoglobin (HGB), and platelets (PLT). Analysis of liver and renal function markers showed that aspartate aminotransferase (AST), alanine aminotransferase (ALT), alkaline phosphatase (ALP), creatinine (CREA), and blood urea nitrogen (BUN) levels remained within normal ranges in both the VHP-GVs and PBS groups (Figure 7e–h).

Hematoxylin and eosin (H&E) staining analysis of main organ sections showed no pathological damage to the heart, liver, spleen, lungs, or kidneys in rats injected with VHP-GVs (Figure 7i). Collectively, these data demonstrate that VHP-GVs exhibit favorable biosafety profiles, making them a promising tool for the in vivo molecular imaging of atherosclerotic plaques.

Long-term histological examination results of organs after repeated injection procedures showed that tissue HE staining analysis on Day 90 post-injection indicated that no pathological damage was seen in the heart, liver, spleen, lungs, and kidneys of rats receiving VHP-GVs injections as compared to the PBS control group (Figure S10).

## 3. Discussion

Atherosclerosis is a multifactorial disease characterized by the accumulation of lipids and inflammatory cells within the arterial wall, leading to the formation of plaques that are prone to rupture [35]. Early identification and precise characterization of these plaques are crucial for effective intervention and management [8]. Driven by the need for non-invasive imaging of atherosclerotic plaques, ultrasound molecular imaging has been a promising strategy for plaque composition and stability [36]. In this study, we developed the acoustic probe VHP-GVs which can specially target VCAM-1, an early inflammatory marker overexpressed in vulnerable plaques, offering a novel ultrasound imaging tool for the early detection of vulnerable plaques.

Different from the previous reports, the biosynthetic GVs derived from *Halobacterium salinarum* NRC-1 were utilized as a platform for design of targeted probes. These GVs, composed of structural proteins GvpA and GvpC, possess several advantageous properties, including (1) their small size (100–200 nm) allows them to penetrate the vascular endothelium and reach the interior of plaques [37,38,39], (2) their biocompatibility minimizes potential toxicity and adverse effects [40], and (3) their uniform size distribution and colloidal stability of these nanobubbles, relative to the SonoVue particles, underscore their potential as highly effective UCAs [28]. Due to immunogenicity or side effects, which are caused by the protein shell contained in GVs being easily cleared by macrophages of the reticuloendothelial system (RES), our research team members have previously completed experiments on the immunogenicity of GVs and PEG-GVs in vitro and in vivo. Modifying with PEG reduces the immunogenicity of biosynthetic bubbles. The research shows that surface modification with PEG greatly reduces the RES’s uptake of nanoparticles and prolongs the circulation time of nanoparticles in the body. Meanwhile, surface modification through PEG can also shield the surface antigens of nanoparticles, reducing the occurrence of immune reactions [41]. Given that the modification by PEG can effectively improve the circulation duration by avoiding recognition and clearance by the mononuclear phagocyte system [24], in this study, GVs were PEGylated to prolong their in vivo circulation time over 10 min, increasing the likelihood of targeting and binding to the plaque site. Moreover, the modification by PEG endows GVs with the electrical neutrality surface, minimizing negative electrical interactions between GVs and maintaining their stability. It is consistent with the previous research findings [41].

Prolonged retention of conventional contrast agents on the plaque surface is challenging due to the shear force exerted by high-speed blood flow. Vulnerable plaques [42,43,44,45,46] exhibit a more active inflammatory response than stable plaques [44,45,46,47], driven by the upregulation of adhesion molecules on the surface of vascular endothelial cells on plaque surface such as VCAM-1 by inflammatory cytokines like TNF-α [42,43]. Yan et al. [17] developed the triple-targeted microbubble UCAs labeled with multiple inflammatory markers (VCAM-1, ICAM-1, and P-selectin). Similarly, Wang et al. [9] applied chemically synthesized anti-VCAM-1 nanoscale UCAs for ultrasound molecular imaging of vulnerable atherosclerotic plaques in a rabbit model. Both studies confirmed the feasibility of VCAM-1 as a molecular target and demonstrated the diagnostic value of ultrasound molecular imaging for vulnerable plaques. By conjugating the VHPKQHR peptide to the GVs, we designed the VHP-GVs which can specifically bind with VCAM-1, an early inflammatory marker that is upregulated in vulnerable plaques. Our in vitro and in vivo experiments demonstrated that VHP-GVs exhibited superior targeting ability compared to the Con-GVs. The enhanced binding affinity of VHP-GVs to VCAM-1 was confirmed through flow cytometry and confocal microscopy, showing significant adhesion to inflamed endothelial cells. This targeting strategy enabled the selective accumulation of VHP-GVs at sites of inflammation, thereby enhancing the contrast signals and improving the detection of vulnerable plaques.

It should be pointed out that most of traditional UCAs, such as SonoVue, are limited by their larger particle size (typically in the micrometer range), which restricts them to penetrate the vascular endothelium and to enter the plaque [18,48]. Also, the flow characteristics of SonoVue closely resemble those of red blood cells in vivo, with a tendency to migrate toward the axial center of the blood vessel [49]. This behavior reduces microbubble contact with the endothelium [50]. As a result, conventional microbubbles can only outline the contour of the plaque within the vessel lumen, providing limited information on plaque composition and stability [51]. In addition, some small plaques are missed due to angiographic signal spillage. In contrast, the nanoscale GVs used in our study demonstrated superior performance in terms of plaque penetration and contrast enhancement. GVs facilitate to produce an enhanced contrast imaging of the vascular wall, and the smaller size of GVs allows them to more effectively interact with the endothelial surface and infiltrate into the plaque. In our in vivo ultrasound imaging studies, VHP-GVs produced stronger and more prolonged contrast signals at the plaque sites. As time went on, only the plaque area showed the significantly enhanced contrast signals, indicating their potential of VHP-GVs for precise and sensitive detection of vulnerable plaques. This finding was further supported by the histological analysis of the plaques, which showed high expression of VCAM-1 and the presence of inflammatory cells, confirming the characteristics of vulnerable plaques [42,43,44] and the targeting specificity of VHP-GVs. Notably, VHP-GVs did not produce significantly enhanced contrast signals for stable plaques when comparing the Con-GVs. The possible reasons may attribute to the following aspects. (1) Calcified plaques represent a relatively stabilized stage in plaque progression, characterized by the gradual reduction in lipid core components and proportional increase in calcified deposits [44]. The literature demonstrated that macrophages within calcified plaques undergo phenotypic switching from pro-inflammatory M1 to anti-inflammatory M2 polarization [46,47]. (2) Calcified plaques have reduced levels of inflammatory factors [45], leading to a relative reduction in the binding of VHP-GVs to the target VCAM-1.

The findings of this study open up new avenues for ultrasound molecular imaging of early atherosclerotic plaque. However, several challenges remain to be addressed before the clinical translation of VHP-GVs. Firstly, long-term side-effect studies are needed to evaluate the potential chronic effects of GVs. Secondly, further optimization of the GVs’ production and purification processes is required to ensure consistent quality and stability. Thirdly, the exploration of imaging protocols is still be needed for enhancing the specificity and sensitivity of GVs. Despite these challenges, biosynthetic nanobubbles hold promise for playing a pivotal role in the early detection, treatment monitoring, and prognostic assessment of atherosclerotic plaques.

## 4. Materials and Methods

### 4.1. Materials

The *Halobacterium salinarum* NRC-1 strain and HUVECs (Source: CRL-1730) were procured from the American Type Culture Collection (ATCC, Rockefeller, MD, USA). SonoVue was acquired from Bracco Suisse SA (Cadempino, Switzerland). Anti-VCAM-1 polyclonal rabbit antibody was obtained from Abways (Shanghai, China). Antibodies against α-smooth muscle actin (α-SMA), CD31, and F4-80, Alexa Fluor 488-labeled goat anti-rabbit IgG, and HRP-labeled goat anti-rabbit IgG were purchased from Servicebio (Wuhan, China). FITC-labeled goat anti-rabbit IgG was sourced from Nakasugi Jinqiao (Beijing, China). MAL-PEG_2000_-NHS (PEG) was acquired from Pengshuo Biologicals (Wuhu, China). The VHPKQHR peptide and MEHFRWG peptide were synthesized by J&L Biochemical (Shanghai) Co., Ltd. (Shanghai, China). Four-week-old male healthy SD rats (weighing approximately 250 g) (including subsequent atherosclerotic plaque model preparation or normal diet feeding) were all sourced from Shanghai Zhan Diem Biotech Co., Ltd. (Shanghai, China). A high-fat diet (D12108C, containing 40% fat and 1.25% cholesterol) for rats was purchased from Kibi Yutai (Beijing) Biotechnology Co., Ltd. (Beijing, China). Research manuscripts reporting large datasets that are deposited in a publicly available database should specify where the data have been deposited and provide the relevant accession numbers. If the accession numbers have not yet been obtained at the time of submission, please state that they will be provided during review. They must be provided prior to publication.

### 4.2. Preparation of Gas Vesicles

GVs were isolated and purified from *Halobacterium salinarum* NRC-1. The bacteria were cultured in ATCC medium (1 L containing 3.0 g tryptone, 5.0 g yeast extract, 250 g NaCl, 20 g magnesium sulfate heptahydrate, 3 g trisodium citrate, and 2 g potassium chloride) supplemented with 0.1 mL of micronutrients (per 200 mL: 1.32 g zinc sulfate heptahydrate, 0.34 g manganese sulfate, 0.82 g ammonium ferrous sulfate, and 0.14 g copper sulfate pentahydrate) for 7–10 days at 37 °C with constant shaking at 220 rpm. The culture was then transferred to a separation funnel and left undisturbed for 1–2 weeks to allow GV-containing bacteria to float. The floating layer was collected and lysed using a hypotonic rupture method with TMC lysis buffer (2 L containing 2.42 g Tris, 1.01 g MgCl_2_, 0.58 g CaCl_2_·2H_2_O, pH 7.5). The lysate was centrifuged 3–5 times at 300× *g* for 3 h each at 4 °C to isolate GVs. The concentration of GVs was determined by measuring the optical density at 500 nm (OD_500_) using a full-wavelength spectrophotometer (Scientific Multiskan GO, Thermo Fisher, Waltham, MA, USA). Specifically, OD_500_ 0.5 represents 3 × 10^10^/mL of GV-containing particles, OD_500_ 1.0 represents 6 × 10^10^/mL of GV-containing particles, OD_500_ 1.5 represents 9 × 10^10^/mL of GV-containing particles, OD_500_ 2.0 represents 1.3 × 10^11^/mL of GV-containing particles, OD_500_ 2.5 represents 2.5 × 10^11^/mL of GV-containing particles, OD_500_ 3.0 represents 1.0 × 10^12^/mL of GV-containing particles, and OD_500_ 3.5 represents 9 × 10^12^/mL of GV-containing particles.

For PEGylation, 10 mL of GVs (OD_500_ = 3.5) were incubated with 300 mg of MAL-PEG_2000_-NHS at 4 °C overnight under gentle mixing conditions. PEGylation was achieved through esterification, wherein the N-hydroxysuccinimide (NHS) group of PEG reacted with amino groups on the GVs. Unbound PEG molecules were removed by dialysis, and PEG-GVs were concentrated by centrifugation. Subsequently, 10 mL of PEG-GVs (OD_500_ = 3.5) were incubated with 1 mg/mL of the VHP peptide or control peptide at 4 °C overnight with gentle shaking. The FITC -labeled peptides (FITC-VHP or FITC-Con) were used to obtain the FITC-labeled GVs. Unbound peptides were removed by dialysis, obtaining the final products: VHP-GVs, Con-GVs, FITC-labeled VHP-GVs (FITC-VHP-GVs), and FITC-labeled Con-GVs (FITC-Con-GVs). SonoVue, supplied as one vial containing 59 mg of SF_6_ gas and 25 mg of lyophilized powder, was reconstituted as a microbubble suspension with an SF_6_ concentration of 45 μg/mL by adding 5 mL of saline (0.9% NaCl) immediately prior to use.

### 4.3. Characterization of Gas Vesicles

For morphological analysis, GVs and *Halobacteria* were diluted and deposited onto a copper grid, negatively stained with 2% phosphotungstic acid, and air-dried at room temperature. The morphology of *Halobacteria* and the GVs released from the bacterial lysis were examined using TEM (Hitachi H-7650, Hitachi Limited, Tokyo, Japan). For SonoVue, its morphology and distribution were visualized using an inverted fluorescence microscope (MF52-N, Mshot, Guangzhou, China) after appropriate dilution. The particle size, PDI, and zeta potential of various GVs (including GVs, PEG-GVs, Con-GVs and VHP-GVs) and SonoVue were measured using a Zetasizer Nano S90 (Malvern Ltd., Malvern, UK) during the preparation stages. All samples were diluted to the appropriate concentration with PBS for various GVs or saline for SonoVue at 4 °C. Each sample was measured in triplicate. Stability assessment of GVs was performed by measuring particle size, PDI, and zeta potential at various time points post-preparation (0, 3, and 7 days). In addition, further we evaluated the stability of VHP-GV stored in PBS, cell culture medium, and serum for 3 days and 7 days.

### 4.4. In Vitro Ultrasound Contrast Imaging of GVs

To evaluate the in vitro contrast imaging capabilities of GVs, agar gel phantoms were prepared by boiling 1% (*w*/*v*) agar powder with pure water. GVs, Con-GVs, and VHP-GVs, at various concentrations (OD_500_ = 0.5, 1.0, 1.5, 2.0, 2.5, 3.0, and 3.5), were added to individual agar wells. Ultrasound contrast imaging was performed using a clinical diagnostic ultrasonography system equipped with an L11-3U linear array probe (Mindray Resona 9, Mindray, Shenzhen, China). Imaging parameters were kept constant throughout the experiment: acoustic power, 5.13%; mechanical index, 0.167; transducer emission frequency, 7.1 MHz; frame rate, 10 frames/s; and gain, 70 dB. Each sample was imaged three times, and the signal intensity was quantified by measuring the gray value of the region of interest (ROI) using Image J software (Image J version1.53t).

### 4.5. Cell Culture and Inflammation Model

HUVECs were cultured in high-glucose Dulbecco’s Modified Eagle Medium (DMEM) supplemented with 10% fetal bovine serum (FBS) and 1% penicillin-streptomycin at 37 °C in a humidified atmosphere containing 5% CO_2_. During the logarithmic growth phase, cells were seeded into 6-well plates at a density of 1 × 10^5^ cells per well. Upon reaching 60–70% confluence, the medium was replaced with serum-free high-glucose DMEM, and the cells were cultured for additional 12 h. These 6-well plates were divided into three groups: the blank control group maintained in medium without TNF-α, and two treatment groups exposed to TNF-α at a final concentration of 40 ng/mL for either 8 or 24 h. After treatment, cells were harvested by trypsinization, washed with PBS, and fixed with 4% paraformaldehyde for 10 min at room temperature. Single-cell suspensions were incubated with anti-VCAM-1 antibody at 4 °C overnight, followed by staining with FITC-conjugated goat anti-rabbit IgG for 20 min at 4 °C in the dark. Flow cytometry was performed to quantify the expression of VCAM-1 proteins on the cell surface. We repeated the operation three times. The optimal TNF-α stimulation time for subsequent experiments was determined based on these results.

### 4.6. In Vitro Cell Adhesion Assay of VHP Peptides

HUVECs were seeded in 6-well plates and stimulated with TNF-α at a concentration of 40 ng/mL for the optimal duration determined from the previous experiments. After stimulation, cells were fixed with 4% paraformaldehyde for 10 min at room temperature. The VHP and control peptides, both conjugated with an FITC fluorescent marker, were prepared as 10 µg/mL solutions in PBS. The FITC-labeled VHP or FITC-labeled control peptides were added to these cells, with a blank group receiving only PBS as the control. All the samples were incubated at 4 °C in the dark for 15 min to facilitate peptide binding. After incubation, the cells were washed via centrifugation in PBS and analyzed using flow cytometry to assess the binding capacity of the VHP and control peptides to VCAM-1 expressed on the cell surface. We repeated the operation three times.

### 4.7. In Vitro Cell Adhesion Assay of VHP-GVs

HUVECs were seeded in 24-well plates and stimulated with TNF-α at a concentration of 40 ng/mL for the optimal duration determined from prior experiments. Then, the cells were fixed with 4% paraformaldehyde for 10 min at room temperature, permeabilized with 0.5% Triton X-100 for 10 min, and blocked with 3% bovine serum albumin (BSA) for 1 h. Nuclei were stained with 4′,6-diamidino-2-phenylindole (DAPI). Next, the cell adhesion with Con-GVs and VHP-GVs was evaluated. For the blocking group, cells were pre-incubated with 1 µg/mL of non-fluorescent VHP peptide for 1 h to blocking VCAM-1 binding sites. Subsequently, 200 µL of PBS was added to the blank control group wells, and 200 µL of FITC-VHP-GVs or FITC-Con-GVs was added to these cells. All the samples were incubated at room temperature in the dark for 15 min. Unbound VHP-GVs or Con-GVs were removed by washing the cells 3–5 times with PBS. Fluorescence images were acquired using a laser confocal microscope (A1R, Nikon, Tokyo, Japan), and fluorescence intensity was quantified using Image J software (Image J version1.53t).

### 4.8. In Vivo Ultrasound Molecular Imaging

The animal study protocol was approved by the institutional review board of the Shenzhen Institute of Advanced Technology, Chinese Academy of Sciences (approval number: SIAT-IACUC-211108-HCS-YF-A2079, approval date: 8 November 2021). A total of 35 healthy male SD rats (weighing about 250 g) at 4 weeks of age were randomly divided into two groups. The control group (15 rats) received a normal diet without mechanical manipulation for four weeks. The experimental group (20 rats) underwent balloon injury surgery on the left common carotid artery. After surgery, they continued to be fed a high-fat diet (D12108C feed) for four weeks [52,53,54]. The left common carotid artery was examined using two-dimensional ultrasound to assess lumen occupancy and blood flow patency to determine the effect of plaque formation. A total of 16 SD rat models of atherosclerotic plaques were obtained (four with unobstructed blood flow in the vessels and no abnormal echoes in the lumen were considered as failures in plaque model preparation) and randomly subjected to in vivo injection experiments. Subsequently, based on the initial two-dimensional ultrasound assessment of plaque formation to confirm successful model preparation, the plaque manifestations varied. The 16 experimental group plaque model rats were divided into the vulnerable plaque group (characterized as early vulnerable plaques with low and mixed echoes in two-dimensional ultrasound mode, 9 rats) and the stable plaque group (characterized as calcified plaques with strong echoes in two-dimensional ultrasound mode, 7 rats) according to the initial ultrasound examination results. Six rats were randomly selected from each group for their respective experimental studies. Ultrasonic imaging of the SD rats was performed using a clinical ultrasound diagnostic system (Mindray Resona 9). Briefly, the SD rats were anesthetized with 1% isoflurane delivered at 2 L/min via inhalation, and body temperature was maintained using a heating pad in the supine position. The neck skin was exposed, and an L11-3U linear array probe was used for two-dimensional ultrasonography. The imaging angle was adjusted to obtain clear views of the left common carotid artery, followed by switching to contrast-specific ultrasonography mode using the same imaging parameters as described for in vitro studies. For the in vivo imaging, Con-GVs and VHP-GVs were administered to random rats with atherosclerotic plaques in the left common carotid artery at varying concentrations (OD_500_ = 2.0, 2.5, 3.0, and 3.5) to evaluate their imaging effects. The optimal concentration and injection sequence were determined on the basis on imaging outcomes. To compare imaging differences between injection sequences, the same concentration of PEG-GVs was injected into the same SD rat three times, with a 30 min interval between two injections to ensure complete metabolic clearance of the previously injected GVs and eliminate residual circulating particles. The same operation was repeated on three rats to obtain three sets of data for statistical analysis. We analyzed the differences in ultrasound imaging between different injection times by injecting the same concentration and same dose of PEG-GVs into the same SD rat three times.

Based on these results, 300 µL of VHP-GVs, Con-GVs, and SonoVue (45 µg/mL) were injected at the optimized concentrations, and contrast imaging of the vessels was performed for 10 min to assess differences in signal intensity of overall and plaque-localized contrast signals within the vessel lumen. From the acquired 10 min video, one frame per second was extracted. The area of plaque perfusion at peak intensity was manually defined as the ROI. Ultrasound images were processed and quantified using Image J software (Image J version1.53t), and the average gray value of each frame was measured to generate a time-intensity curve.

### 4.9. Histological Examination

The presence of vulnerable plaques in SD rats was histologically confirmed. The left common carotid arteries were harvested from three SD rats with plaques, dissected, and fixed in 4% paraformaldehyde for 48 h. Frozen sections of the vascular luminal plaque lesions were prepared for histological analysis. Tissue sections were subjected to immunofluorescence staining for VCAM-1, H&E staining, and Masson’s trichrome staining to evaluate the histological features of the plaques. Additionally, immunohistochemical staining was also performed using antibodies against F4/80, α-SMA, and CD31 within the plaque to characterize the composition and structural features of the atherosclerotic plaques.

### 4.10. Toxicity Assay

#### 4.10.1. Cell Viability Assay

The viability of HUVECs was assessed using the CCK-8 (Beyotime, Shanghai, China) assay. HUVECs were seeded in a 96-well plate with 5 × 10^4^ cells per well. Upon reaching 60–70% confluence, 100 µL of DMEM containing VHP-GVs at various concentrations (OD_500_ = 0.5, 1.0, 1.5, 2.0, 2.5, 3.0, and 3.5) was added to the wells. Cells were incubated for 12 or 24 h, respectively. After incubation, cells were rinsed with PBS, and 100 µL of DMEM containing 10 µL of CCK-8 reagent was added to each well. The plates were then incubated for 1 h at 37 °C. Cell viability was determined by measuring the absorbance at 450 nm using a microplate reader. Viability was calculated using the following formula:

Viability (%) = [(OD_GVs_ + OD_blank_)/(OD_control_ + OD_blank_)] × 100%,

where OD_GVs_ represents the absorbance of cells treated with VHP-GVs, OD_control_ represents the absorbance of untreated control cells, and OD_blank_ represents the absorbance of the blank medium.

#### 4.10.2. Hemolysis Assay

A hemolysis assay was performed using fresh blood obtained from SD rats. Blood was collected via orbital puncture and centrifuged at 1500 rpm for 15 min to isolate erythrocytes, which were subsequently washed twice with PBS. Distilled water (ddH_2_O) was used as the positive control for 100% hemolysis, while PBS as the negative control for 0% hemolysis. Erythrocytes were incubated with Con-GVs and VHP-GVs at various concentrations (OD_500_ = 0.5, 1.0, 1.5, 2.0, 2.5, 3.0, and 3.5) for 3 h at 37 °C. Following incubation, samples were centrifuged at 13,000 rpm for 15 min, and the extent of hemolysis was quantified by measuring the absorbance of the supernatant at 540 nm using a microplate reader. The percentage of hemolysis was calculated using the following formula:

Hemolysis (%) = [(Absorbance_sample_ − Absorbance_PBS_)/(Absorbance_H2O_ − Absorbance_PBS_)] × 100%,

where Absorbance_sample_ represents the absorbance of the supernatant from samples incubated with VHP-GVs or Con-GVs, Absorbance_PBS_ represents the absorbance of the negative control (PBS), and Absorbance_H2O_ represents the absorbance of the positive control (distilled water).

#### 4.10.3. In Vivo Biosafety Assay

The in vivo biosafety of VHP-GVs was assessed through hematological analyses (complete blood count and blood biochemistry) and histological examination of major organs. Nine healthy SD rats were randomly divided into three groups with three rats each group: the PBS control group, the GVs group, and the VHP-GVs group. Each group received a single intravenous injection of 300 µL of PBS, GVs, or VHP-GVs, respectively. Blood samples were collected from the orbital sinus at three time points: pre-injection, 1 day post-injection, and 7 days post-injection. Complete blood counts and biochemical markers of liver and renal function were analyzed. The complete blood count analysis included measurements of WBCs, RBCs, PLT, and HGB. Hepatic function was assessed using markers such as aspartate AST, ALT, and ALP. Renal function was evaluated based on serum CREA and BUNlevels. On the seventh day post-injection, the rats were euthanized and major organs (heart, liver, spleen, lung, and kidney) were harvested. The tissues were fixed in 4% paraformaldehyde, embedded in paraffin, sectioned, and stained with H&E for histological evaluation.

#### 4.10.4. Repeat-Dose and Long-Term Organ Retention Assessment

Six healthy SD rats were randomly divided into two groups: the PBS group and the VHP-GVs group, with three rats in each group. Rats were injected with 300 µL of PBS and VHP-GVs in the tail vein once every seven days, respectively. Both injections were administered six times. Rats were sacrificed 90 days after the first injection, and their main organs, including the heart, liver, spleen, lungs, and kidneys, were collected. The tissues were fixed with 4% paraformaldehyde, embedded in paraffin, and stained with HE.

### 4.11. Statistical Analysis

Statistical analyses were performed using SPSS version 22.0. If the quantitative variable follows a normal distribution and the homogeneity of variance test indicates homogeneity of variance between groups, then the comparison between two groups is conducted using an independent sample *t*-test, and the comparison among multiple groups is conducted using one-way analysis of variance (ANOVA) followed by a Bonferroni multiple comparison test. Quantitative data are presented as mean ± standard deviation (SD). Graphical representations were generated using GraphPad Prism 8.0 software. The intra-observer repeatability was assessed three times, and three observers independently analyzed the images. Intraclass correlation coefficients (ICCs) were used to analyze the consistency between observers and within observers to repeatability tests. Statistical significance was defined as *p* < 0.05, and high significance was defined as *p* < 0.01.

## 5. Conclusions

In summary, we developed the nanoscale VHP-GVs on the basis of biosynthetic GVs from *Halobacterium salinarum* NRC-1. Our results confirm that VHP-GVs can serve as novel targeted probe with markedly superior imaging performance in atherosclerotic vulnerable plaques, facilitating precise diagnosis of atherosclerosis, especially for vulnerable plaques. Our study offers a new tool for precise diagnosis of vulnerable plaques, with potential for clinical translation and application.

## Data Availability

The original contributions presented in this study are included in the article/Supplementary Materials. Further inquiries can be directed to the corresponding author.

## Supplementary Materials

The following supporting information can be downloaded at: https://www.mdpi.com/article/10.3390/ph18091285/s1, Figure S1: The process of extracting GVs from Halo; Figure S2: Stability assessment of GVs; Figure S3: Flow cytometry gating strategy for expression levels of VCAM-1 on the surface of HUVECs after 8 h and 24 h by TNF-α stimulation; Figure S4: Flow cytometry gating strategy for VCAM-1 targeting experiments; Figure S5: Contrast-enhanced ultrasound imaging of different concentrations of Con-GVs and VHP-GVs at different times after in vivo injection; Figure S6: Time-intensity curves of intravascular contrast-enhanced ultrasound signals within 500 s post-injection of Con-GVs and VHP-GVs at different concentrations; Figure S7: Serial contrast-enhanced ultrasound imaging in the same SD rat following three sequential injections of PEG-GVs at identical concentrations; Figure S8: Time-intensity curves of intravascular contrast-enhanced ultrasound signals after three consecutive injections of the same concentration of PEG-GVs; Figure S9: Time-intensity curves of intravascular contrast-enhanced ultrasound signals and the blood half-life of the contrast agent in the body (estimated based on the ultrasound signal intensity of the contrast agent) after injections of VHP-GVs; Table S1: Repeatability test of ultrasound signal intensity for PEG-GVs, Con-GVs, and VHP-GVs; Figure S10: Representative H&E-stained sections of major organs (heart, liver, spleen, lungs, kidneys) from rats 90 days after repeated injections multiple times of PBS or VHP-GVs.
