# Supplementary material for: Early Detection of Vulnerable Plaques Using Targeted Biosynthetic Nanobubbles
Source: Pharmaceuticals (Basel). 2025 Aug 28;18(9):1285. doi: 10.3390/ph18091285 (PMC12472982; doi:10.3390/ph18091285)
Supplement: Supplementary file 1 [file pharmaceuticals-18-01285-s001.zip › pharmaceuticals-3770291-supplementary.pdf]

Supplementary Material

Early Detection of Vulnerable Plaques Using Targeted Biosynthetic Nanobubbles

Yan Wang [1], Huang Yin [2], Rui Zhang [1], Dan Yu [1], Jieqiong Wang [3], Tingting Liu [4], Xiong Shen [4,5], Li Xue [1,6,*] and Fei Yan [7,*]

1 Department of Cardiovascular Ultrasound, the Fourth Affiliated Hospital of Harbin Medical University, Harbin 150001, China; hydwyan@163.com (Y.W.); zhangr_1995@126.com (R.Z.); 15005054415@163.com (D.Y.)

2 The Fifth Affiliated Hospital, Sun Yat-sen University, Zhuhai 519000, China; huangyin3@sysu.edu.cn

3 Department of Rehabilitation Medicine, Huashan Hospital, Fudan University, Shanghai 201206, China; wangjieqiong.123@foxmail.com

4 Department of Ultrasound, The Second People's Hospital of Shenzhen, The First Affiliated Hospital of Shenzhen University, Shenzhen 518061, China; ltingting949@gmail.com (T.L.); shenxiong2023@126.com (X.S.)

5 Ultrasonic Medicine, Graduate School, Guangxi University of Chinese Medicine, Nanning 530200, China

6 Heilongjiang Provincial Molecular Medicine Engineering Technology Research Center, Harbin 150001, China

7 State Key Laboratory of Quantitative Engineering Biology, Shenzhen Institute of Synthetic Biology, Shenzhen Institutes of Advanced Technology, Chinese Academy of Sciences, Shenzhen 518055, China

* Correspondence: toxueli@163.com (L.X.); fei.yan@siat.ac.cn (F.Y.); Tel.: +86-13394608900 (L.X.); +86-755-8639-2284 (F.Y.)

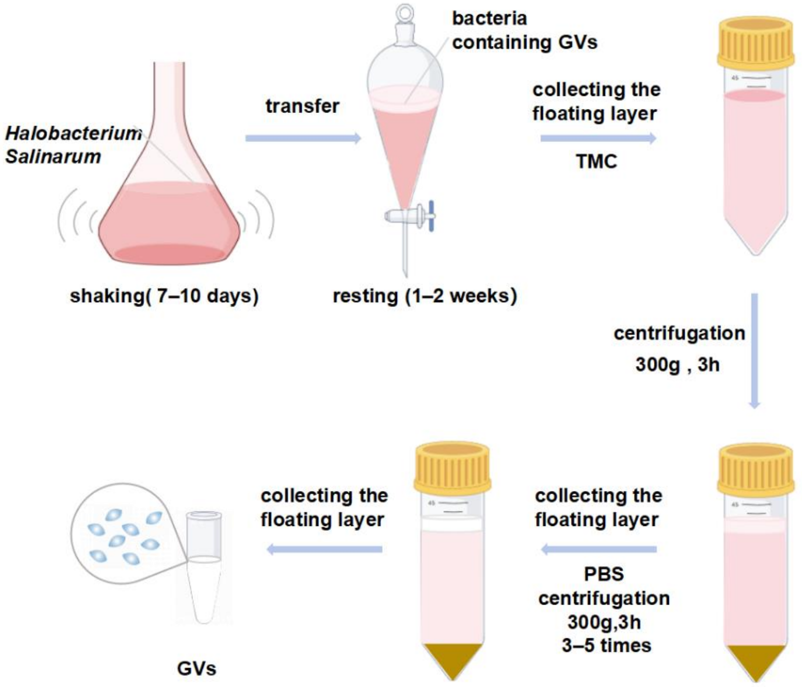

Figure S1. The process of extracting GVs from Halo. The bacteria were cultured in ATCC medium for 7–10 days at 37°C with constant shaking at 220 rpm. The mature liquid of Halo bacteria showed the color of strawberry milk. The bacterial solution was transferred to a pear-shaped liquid separation funnel for 1–2 weeks for static stratification, and the upper layer was a pink and white ring layer, which was the dominant bacterial body with GVs structure. Separate and collect the surface floating layer with TMC lysis buffer for centrifugation in a 50ml centrifuge tube. The lysate was centrifuged 3–5 times at 300g for 3 h each at 4°C to isolate GVs.

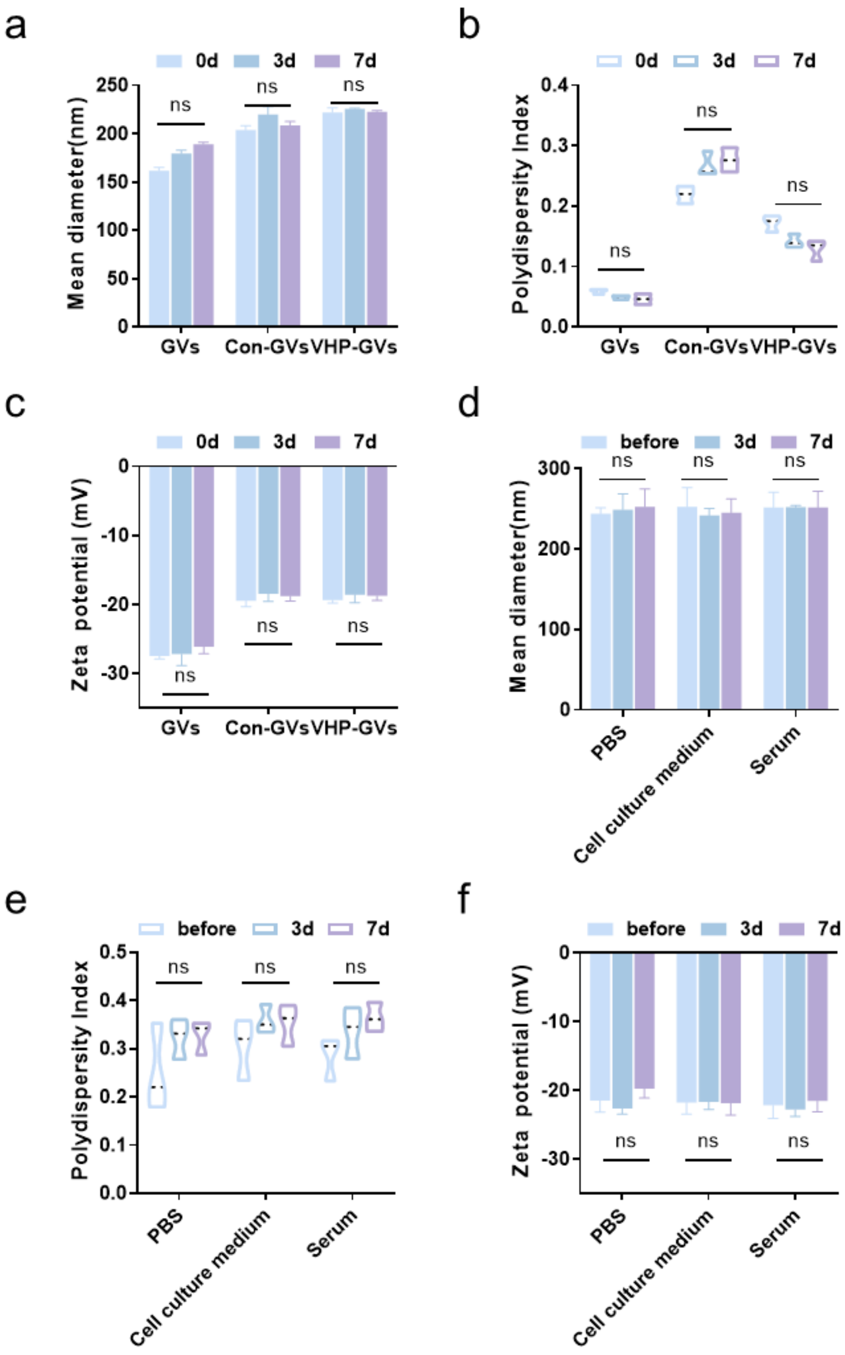

Figure S2. Stability assessment of GVs. (**a**) particle size, (**b**) PDI, and (**c**) zeta potential of GVs, Con-GVs, and VHP-GVs at various time points post-preparation (0, 3, and 7 days) in PBS. (**d**) particle size, (**e**) PDI, and (**f**) zeta potential of VHP-GVs in PBS, cell culture medium and serum for 3 days and 7 days. Data are presented as mean ± standard deviation from three independent experiments, and ns for no statistical significance.

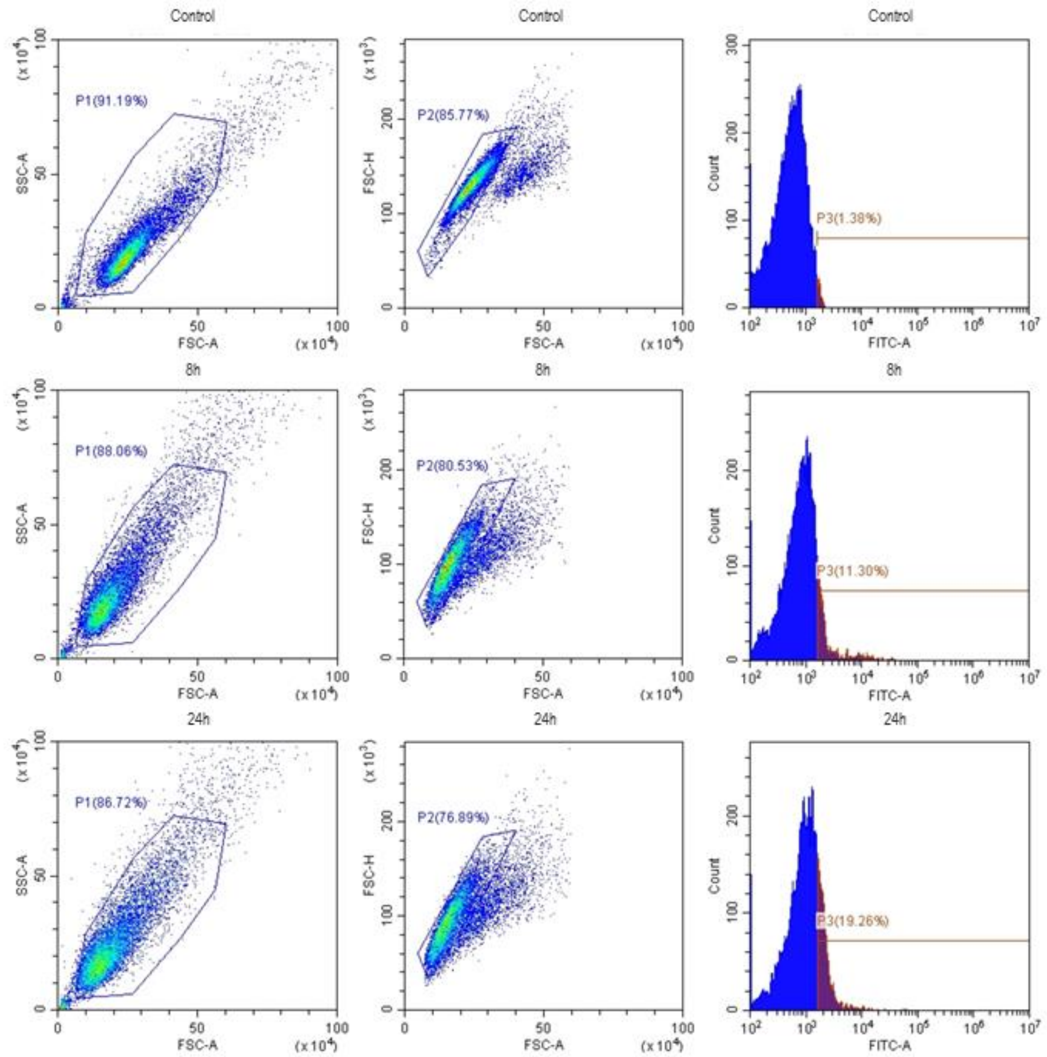

Figure S3. Flow cytometry gating strategy for expression levels of VCAM-1 on the surface of HUVECs after 8 h and 24 h by TNF-α stimulation. Debris was excluded based on SSC-A vs FSC-A. Single cells were selected from FSC-H vs FSC-A. FITC positive cells were defined relative to a cell-only control sample.

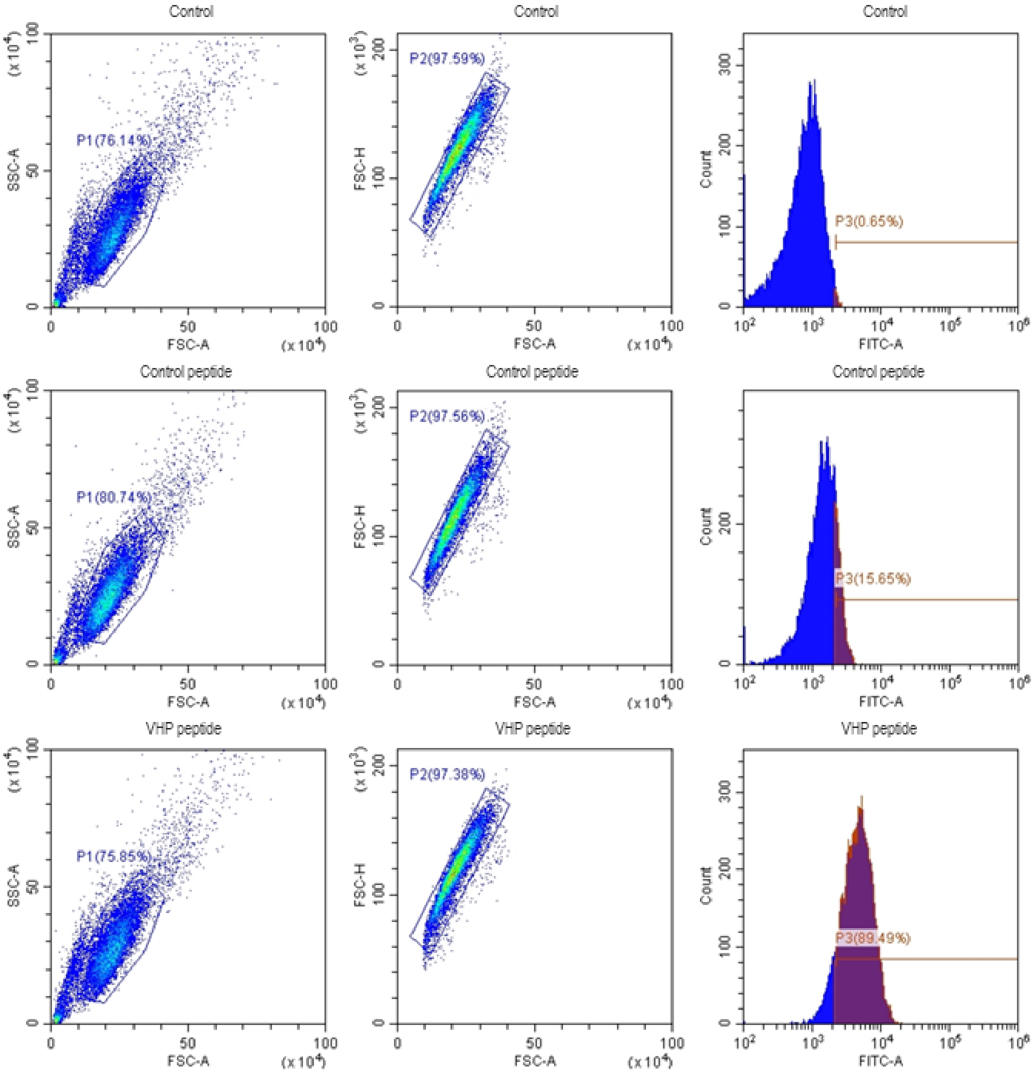

Figure S4. Flow cytometry gating strategy for VCAM-1 targeting experiments. Debris was excluded based on SSC-A vs FSC-A. Single cells were selected from FSC-H vs FSC-A. FITC positive cells were defined relative to a cell-only control sample.

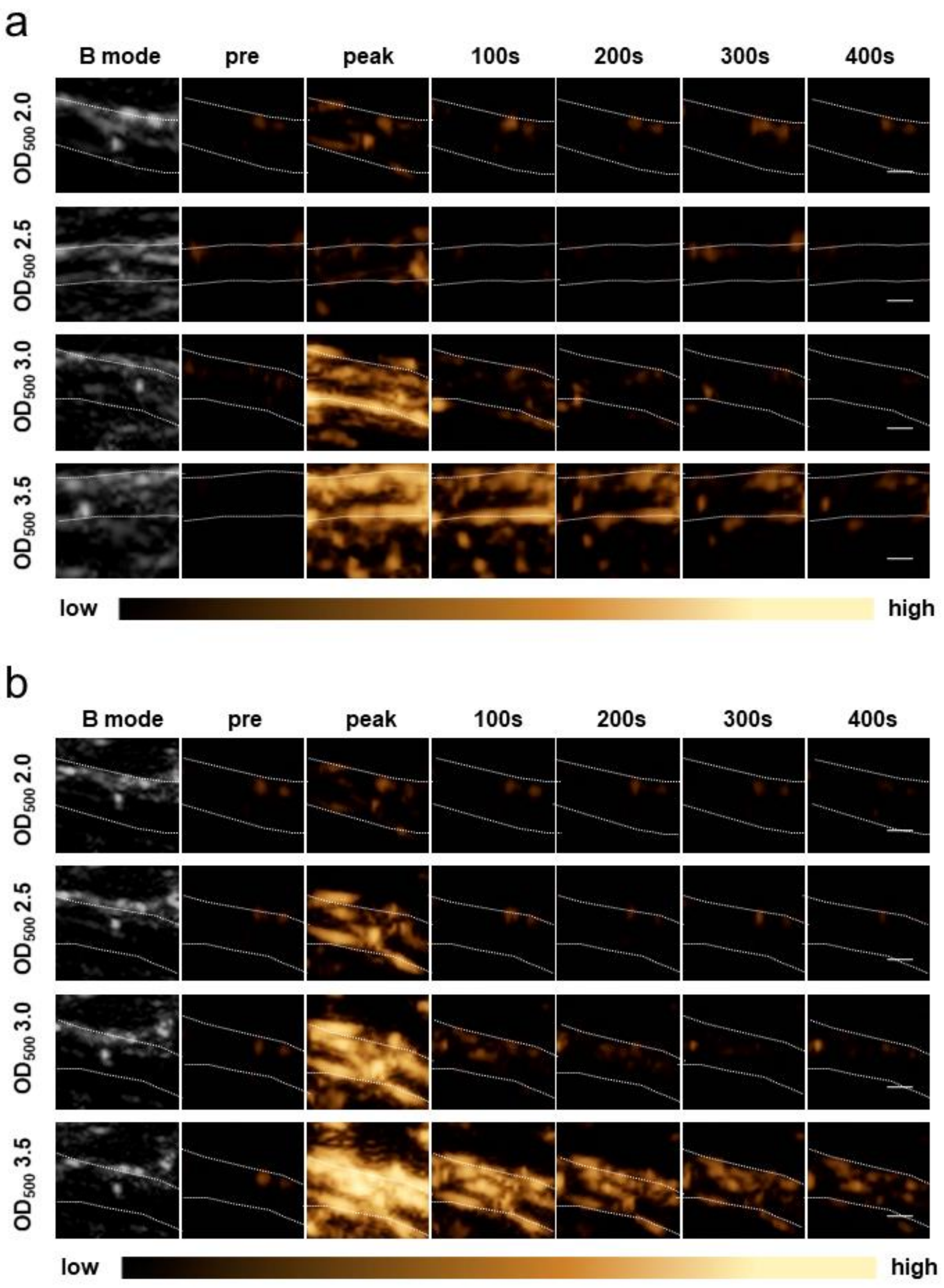

Figure S5. Contrast-enhanced ultrasound imaging of different concentrations of Con-GVs and VHP-GVs at different times after in vivo injection. (**a**) Con-GVs; (**b**) VHP-GVs. The two white dashed lines are used to indicate the vascular wall lumen. Scale bar = 4000 μm.

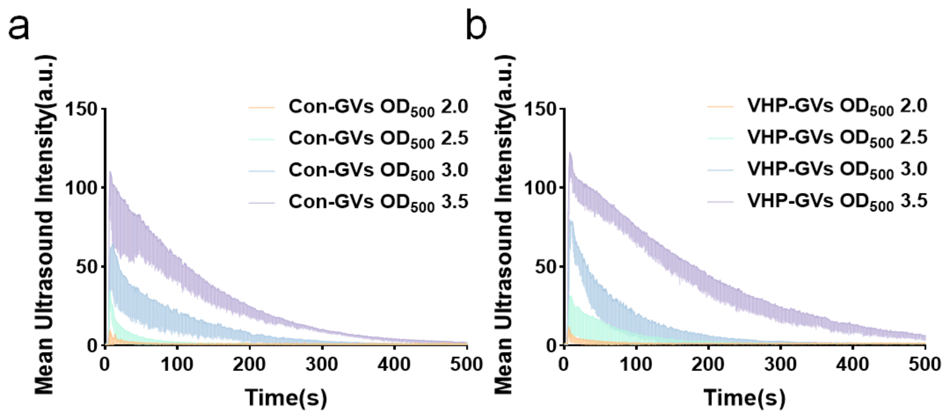

Figure S6. Time-intensity curves of intravascular contrast-enhanced ultrasound signals within 500 seconds post-injection of Con-GVs and VHP-GVs at different concentrations. (**a**) Con-GVs; (**b**) VHP-GVs.

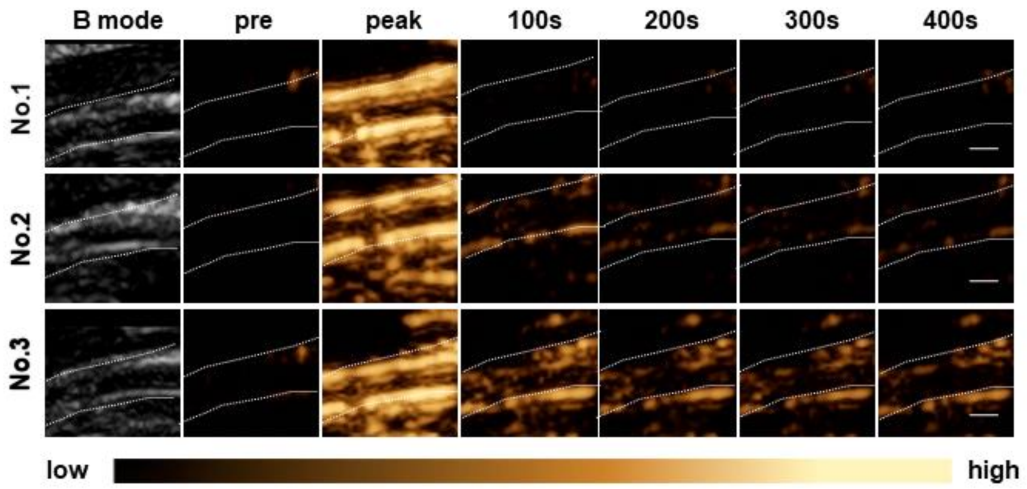

Figure S7. Serial contrast-enhanced ultrasound imaging in the same SD rat following three sequential injections of PEG-GVs at identical concentrations. The two white dashed lines are used to indicate the vascular wall lumen. Scale bar = 4000 μm.

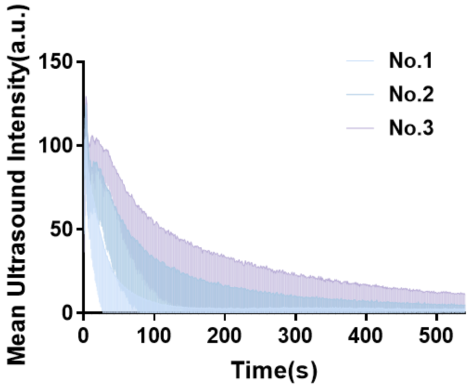

Figure S8. Time-intensity curves of intravascular contrast-enhanced ultrasound signals after three consecutive injections of the same concentration of PEG-GVs.

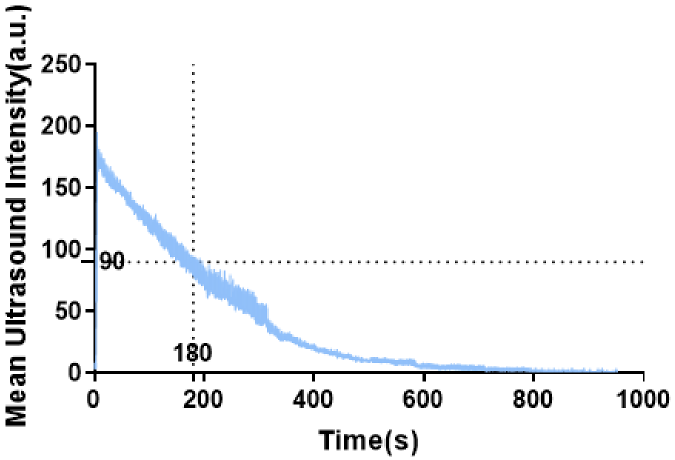

Figure S9. Time-intensity curves of intravascular contrast-enhanced ultrasound signals and the blood half-life of the contrast agent in the body (estimated based on the ultrasound signal intensity of the contrast agent) after injections of VHP-GVs.

Table S1. Repeatability test of ultrasound signal intensity for PEG-GVs, Con-GVs, and VHP-GVs

Index	ICC	95%CI	ICC	95%CI
PEG-GVs	0.970	0.967-0.973	0.942	0.935-0.948
Con-GVs（vulnerable plaques）	0.966	0.962-0.970	0.950	0.945-0.955
VHP-GVs（vulnerable plaques）	0.974	0.971-0.977	0.944	0.938-0.950
Con-GVs（calcified plaques）	0.981	0.978-0.983	0.967	0.963-0.970
VHP-GVs（calcified plaques）	0.975	0.972-0.978	0.963	0.959-0.967

ICC: Intraclass Correlation Coefficient.

CI: Confidence Interval.

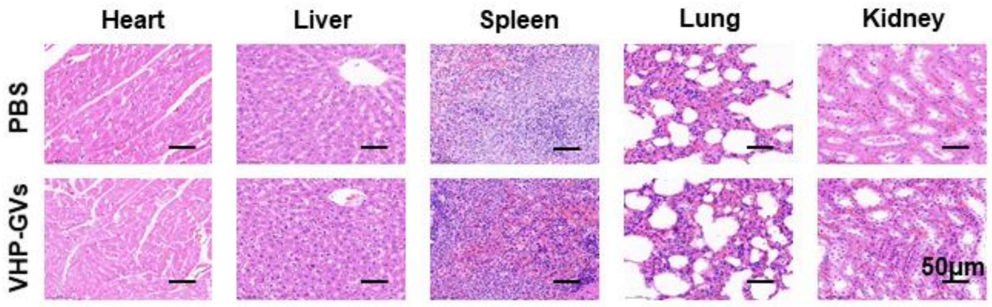

Figure S10. Representative H&E-stained sections of major organs (heart, liver, spleen, lungs, kidneys) from rats 90 days after repeated injections multiple times of PBS or VHP-GVs. Scale bar = 50 μm.
